# Enhancing oncolytic reovirus therapy with proteasome inhibitors in multiple myeloma

**DOI:** 10.1097/MS9.0000000000003256

**Published:** 2026-03-17

**Authors:** Muhammad Rayan Aleem, Khushbakht Baloch, Minahil Shahid, Sk. Sany Talha

**Affiliations:** Jinnah Sindh Medical University, Karachi, Pakistan

## Introduction

Multiple myeloma (MM) is a hematological malignancy characterized by uncontrolled proliferation of cytogenetically different plasma cells. It is the second most common hematological cancer, accounting for approximately 10% of all hematological malignancies, with a slightly higher incidence in men than in women.^[^1,2^]^ Early diagnosis is often delayed due to unspecified symptoms and indolent progression, and patients are mostly diagnosed at a high-risk stage, necessitating aggressive treatment strategies.

Before the 1990s, chemotherapy with alkylating agents was the conventional treatment method, which was replaced by autologous stem cell transplantation in the 1990s.^[^3^]^ However, current treatments for multiple myeloma (MM) include the use of histone deacetylase inhibitors, checkpoint inhibitors, immunomodulatory agents, and oncolytic virotherapy.^[^4^]^ In particular, the use of Reovirus (Pelareorep) has become significant due to its tumor-selective replication along with immunomodulatory effects^[^5^]^. Although these treatments are effective, combination strategies have further enhanced the efficacy of treating MMs.

## Oncolytic virotherapy

Oncolytic virotherapy is a constantly evolving cancer treatment that uses oncolytic viruses (OVs) to destroy cancer cells. Oncolytic virotherapy has shown significant potential for the treatment of various malignancies, including melanomas, pancreatic cancer, colorectal cancer, breast cancer, liver cancer, and hematological malignancies. OVs target cancer cells in distinct ways; for instance, HSV exploits aberrant cell signaling, while adenovirus exploits the tumor by modulating the release of cytokines and production of inflammatory cytokines. Reovirus destroys tumor cells by inducing virus induced apoptosis by activating the RAS pathways^[^6,7^]^.

## Proteasome inhibitors

Proteasome inhibitors are clinically important drugs that have been used in the treatment of MM and cell lymphoma, as they inhibit the catalytically active subunits and lead to tumor cell death. Proteasome inhibitors, including bortezomib, carfilzomib, and ixazomib, have revolutionized the treatment of MM. PIs inhibit the proteasome, leading to disruption of the delicate balance of proteins and suppression of tumor growth and spread. Initially, Bortezomib demonstrated efficacy in treating MM; however, carfilzomib and ixazomib showed improved efficacy and lower toxicity. Incorporation of these inhibitors in the treatment of MM has increased the efficacy of treatment by approximately 60–90%, thus improving the quality of treatment. However, additional efficacy could be obtained by combination of proteosome inhibitors with oncolytic virotherapy^[^8,9^]^.

## Clinical trials of combination therapy

Proteosome inhibitors, checkpoint inhibitors, immunomodulatory agents, and oncolytic virotherapy have shown effective results in the treatment of MM. However, their clinical benefits have been limited by their reduced efficiency in achieving viral replication and delivery to tumor cells.^[^10^]^

**Figure 1.**
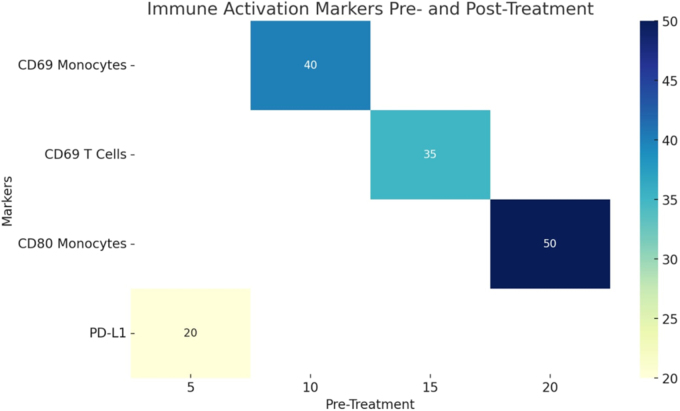
Immune activation markers show significant increases in CD69 monocytes, CD80 monocytes, CD69 T-cells, and PD-L1 expression post-treatment^[^6^]^.

Addressing these challenges, findings from Dona *et al*’s research revealed convincing data on the interactive effects of reovirus therapy with the proteasome inhibitor (PIs) carfilzomib.^[^11^]^ The outcome of this study illustrate that Pls augment reovirus replication in classical monocytes by inhibiting NF-kB mediated antiviral signaling, which is a crucial step in limiting viral spread. In MM, NF-kB is persistently activated and contributes to tumor growth and survival^[^12^]^. Therefore, by blocking NF-kB, Pls suppresses the host cell’s antiviral defense mechanism. This combined method for the treatment of MM not only enhances the anti-tumor effect but also triggers a strong and sustained immune response as seen in Fig. 1.

During the phase 1b clinical trial, the combination of pelareorep and carfilzomib in relapsed and refractory MM patients showed a promising response rate of 70%, revealing the effect of combined treatment. Furthermore, the investigation stresses the fundamental importance of monocytes in assisting viral replication and distribution, establishing PIs as key players in boosting oncolytic virotherapy^[^11^]^.

## Limitations and future directions

Regardless of optimistic results, uncertainty still prevails regarding the synergistic fusion of this treatment with existing treatments, protocols, as phase 1b trial involve a limited number of patients^[^6^]^. Further investigation should emphasize the long-term immunological impacts, refine dosing remedies, and assess the combination efficacy in larger and diverse populations. Additionally, future research should take a closer look at immune checkpoint inhibitors in this treatment strategy, which can help overcome tumor-induced immune suppression and amplify therapeutic potential.

## Conclusion

In conclusion, the curative possibilities of combining PIs with oncolytic reovirus therapy in MM serve as a solid basis for improving outcomes in the treatment of MM. These findings highlight an important step toward improving outcomes in this challenging malignancy and offer a foundation for further clinical and translational exploration. Further studies and clinical trials are required to properly grasp the benefits and incorporate them in the treatment of MM.

## Data Availability

Not applicable.

## Research registration unique identifying number (UIN)

No human subject involved.
